# The effects of hyperoxic and hypercarbic gases on tumour blood flow

**DOI:** 10.1038/sj.bjc.6690330

**Published:** 1999-04

**Authors:** T J Dunn, R D Braun, W E Rhemus, G L Rosner, T W Secomb, G M Tozer, D J Chaplin, M W Dewhirst

**Affiliations:** Departments of Radiation Oncology, Biometry and Medical Informatics, Duke University Medical Center, PO Box 3455, Durham, NC 27710, USA; Department of Physiology, Arizona Health Sciences Center, University of Arizona, Tucson, AZ 85724, USA; Tumour Microcirculation Group, Gray Laboratory Cancer Research Trust, Northwood, Middlesex, HA6 2RJ, UK

**Keywords:** tumour, blood flow, arteriolar diameter, carbogen, carbon dioxide

## Abstract

Carbogen (95% O_2_ and 5% CO_2_) has been used in preference to 100% oxygen (O_2_) as a radiosensitizer, because it is believed that CO_2_ blocks O_2_-induced vasoconstriction. However, recent work suggests that both normal and tumour arterioles of dorsal flap window chambers exhibit the opposite: no vasoconstriction vs constriction for O_2_ vs carbogen breathing respectively. We hypothesized that CO_2_ content might cause vasoconstriction and investigated the effects of three O_2_–CO_2_ breathing mixtures on tumour arteriolar diameter (TAD) and blood flow (TBF). Fischer 344 rats with R3230Ac tumours transplanted into window chambers breathed either 1%, 5%, or 10% CO_2_ + O_2_. Intravital microscopy and laser Doppler flowmetry were used to measure TAD and TBF respectively. Animals breathing 1% CO_2_ had increased mean arterial pressure (MAP), no change in heart rate (HR), transient reduction in TAD and no change in TBF. Rats breathing 5% CO_2_ (carbogen) had transiently increased MAP, decreased HR, reduced TAD and a sustained 25% TBF decrease. Animals exposed to 10% CO_2_ experienced a transient decrease in MAP, no HR change, reduced TAD and a 30–40% transient TBF decrease. The effects on MAP, HR, TAD and TBF were not CO_2_ dose-dependent, suggesting that complex physiologic mechanisms are involved. Nevertheless, when ≥ 5% CO_2_ was breathed, there was clear vasoconstriction and TBF reduction in this model. This suggests that the effects of hypercarbic gases on TBF are site-dependent and that use of carbogen as a radiosensitizer may be counterproductive in certain situations. © 1999 Cancer Research Campaign


*British Journal of Cancer* (1999) **80**(1/2), 117–126
© 1999 Cancer Research Campaign
Article no. bjoc.1998.0330

### The effects of hyperoxic and hypercarbic gases on tumour blood flow

**TJ Dunn[1], RD Braun[1], WE Rhemus[1], GL Rosner[2], TW Secomb[3], GM Tozer[4], DJ Chaplin[4] and MW Dewhirst[1]**

Departments of [1]Radiation Oncology, [2]Biometry and Medical Informatics, Duke University Medical Center, PO Box 3455, Durham, NC 27710, USA; [3]Department of Physiology, Arizona Health Sciences Center, University of Arizona, Tucson, AZ 85724, USA; [4]Tumour Microcirculation Group, Gray Laboratory Cancer Research Trust, Northwood, Middlesex, HA6 2RJ, UK

**Summary** Carbogen (95% $O_2$ and 5% $CO_2$) has been used in preference to 100% oxygen ($O_2$) as a radiosensitizer, because it is believed that $CO_2$ blocks $O_2$-induced vasoconstriction. However, recent work suggests that both normal and tumour arterioles of dorsal flap window chambers exhibit the opposite: no vasoconstriction vs constriction for $O_2$ vs carbogen breathing respectively. We hypothesized that $CO_2$ content might cause vasoconstriction and investigated the effects of three $O_2$–$CO_2$ breathing mixtures on tumour arteriolar diameter (TAD) and blood flow (TBF). Fischer 344 rats with R3230Ac tumours transplanted into window chambers breathed either 1%, 5%, or 10% $CO_2$ + $O_2$. Intravital microscopy and laser Doppler flowmetry were used to measure TAD and TBF respectively. Animals breathing 1% $CO_2$ had increased mean arterial pressure (MAP), no change in heart rate (HR), transient reduction in TAD and no change in TBF. Rats breathing 5% $CO_2$ (carbogen) had transiently increased MAP, decreased HR, reduced TAD and a sustained 25% TBF decrease. Animals exposed to 10% $CO_2$ experienced a transient decrease in MAP, no HR change, reduced TAD and a 30–40% transient TBF decrease. The effects on MAP, HR, TAD and TBF were not $CO_2$ dose-dependent, suggesting that complex physiologic mechanisms are involved. Nevertheless, when ≥ 5% $CO_2$ was breathed, there was clear vasoconstriction and TBF reduction in this model. This suggests that the effects of hypercarbic gases on TBF are site-dependent and that use of carbogen as a radiosensitizer may be counterproductive in certain situations.

**Keywords:** tumour; blood flow; arteriolar diameter; carbogen; carbon dioxide

The presence of hypoxic cells within tumours has a negative impact on several different cancer treatment modalities. In addition to being a well-established cause of resistance to radiation therapy (Thomlinson and Gray, 1955), hypoxia has been shown to have an adverse effect on the in vitro cytotoxic activity of photodynamic therapy (Chapman et al, 1991) and various chemotherapeutic agents, including tumour necrosis factor-α (Sampson and Chaplin, 1994) and adriamycin (Smith et al, 1980). Clinically hypoxic areas have been shown to exist in human tumours and are known to influence patient survival and treatment outcome (Höckel et al, 1993, 1996; Brizel et al, 1996; Nordsmark et al, 1996; Overgaard and Horsman, 1996).

Because of the relative resistance of hypoxic cells to different treatment modalities, and the importance of hypoxia in treatment outcome, much effort has been directed towards overcoming tumour hypoxia. A common current approach has been the breathing of hyperoxic gases, including 100% oxygen ($O_2$) and carbogen (95% $O_2$ and 5% $CO_2$). The rationale for the use of carbogen over 100% $O_2$ is based on the following putative mechanisms: (1) $CO_2$ blocks hyperoxia-induced vasoconstriction, (2) increased $CO_2$ tensions cause a rightward shift of the oxygen–haemoglobin dissociation curve and (3) $CO_2$ has a positive chronotropic effect (Kruuv et al, 1966). Despite these sound theoretical grounds, the inhalation of carbogen has had only moderate success as a radiosensitizer (Siemann et al, 1977; Overgaard, 1989; Rojas, 1991). Although breathing of oxic gases such as carbogen clearly increases the total oxygen content of blood, there may be other complex physiologic interactions which affect the ability of this gas to increase $pO_2$ in solid tumours.

It has long been known that carbogen can inhibit hyperoxia-induced vasoconstriction or even increase blood flow in some tissues, particularly neural tissue like the brain (Kety and Schmidt, 1948) and retina (e.g. Hickam and Frayser, 1966) but there is evidence that carbogen decreases flow in some other tissues (Hampson et al, 1987; Honess and Bleehen, 1995). Furthermore, the vasoactive effects of carbogen appear to be dependent on route of administration, with mechanically ventilated animals responding with vasodilation, and freely breathing animals with vasoconstriction (Kallinen et al, 1991). In addition, recent work has demonstrated that carbogen may induce vasoconstriction in both normal and tumour arterioles (Dewhirst et al, 1996), and can cause simultaneous increases and decreases in both blood flow and $pO_2$ within the same tumours (Powell et al, 1996; Lanzen et al, 1998). Alternatively, a consistent decrease in blood flow was found in subcutaneously implanted sarcoma F tumours in mice during carbogen breathing (Hill et al, 1998). These findings all argue against the proposed mechanism of action of carbogen, at least in some tumours.

Falk and associates (1992) demonstrated that the time course over which carbogen is given significantly impacts its ability to increase oxygenation in tumours. Siemann and co-workers (1977)

*Received 16 January 1998*
*Revised 17 August 1998*
*Accepted 18 August 1998*

*Correspondence to:* MW Dewhirst had previously shown that the timing of carbogen breathing has a direct impact on the outcome of radiation treatment. This phenomenon may be attributable to time-dependent alterations in tumour blood flow (TBF) or it may be partially due to increases in oxygen consumption induced by the use of oxic gases (Dewhirst et al, 1996). Before carbogen can be optimally used as a radiosensitizer, it will be necessary to understand the mechanisms responsible for these complex and variable effects on tumour blood flow and oxygenation.

In a recent study we found that the arterioles of normal tissues and tumours growing in rat dorsal flap window chambers exhibited no vasoconstriction during 100% $O_2$ breathing (Dewhirst et al, 1996). In addition, we found that carbogen breathing tended to cause an initial transient vasoconstriction in both tumour and normal arterioles, although this change was not consistently statistically significant with the small numbers of animals studied. These surprising results inspired us to investigate the effects of hyperoxic and hypercarbic gases in greater detail. We hypothesized that the presence of $CO_2$ in the breathing gas might have vasoconstrictive effects in this model system, and that the level of $CO_2$ in the breathing gas might be directly correlated to the degree of vasoconstriction of the tumour arterioles. In this present study, we performed experiments investigating the effect of breathing gases containing 1% $CO_2$, 5% $CO_2$, or 10% $CO_2$ mixed with oxygen on arteriolar diameter and blood flow in R3230Ac mammary adenocarcinomas growing in the dorsal flap window chamber in Fischer 344 rats.

### MATERIALS AND METHODS

### Animal model

Fischer 344 rats (Charles River Laboratories, Raleigh, NC, USA) were surgically implanted with cutaneous window chambers as described previously (Papenfuss et al, 1979). R3230Ac mammary adenocarcinomas were transplanted onto a fascial plain of subcutaneous tissue at the time of window chamber surgery. Prior to experimentation, the animals were housed individually in an environmental chamber maintained at 34°C and 50% humidity with continuous access to food and water. Experimentation was performed 9–11 days following transplantation. All protocols were approved by the Duke Animal Care and Use Committee.

### Anaesthesia and blood gases

Animals were anaesthetized with sodium pentobarbital (50 mg $kg^{-1}$ intraperitoneally (i.p.)) for all surgical and experimental procedures. Heart rate (HR) and blood pressure were obtained from computerized acquisition (AT-codas, Dataq Instruments, Akron, OH, USA) of femoral arterial waveforms. Blood gas measurements were obtained from 0.2 ml samples of femoral arterial blood. During experimentation, animals were maintained at a rectal temperature of 36–37°C using a thermostatically controlled blanket (Model 50-7503 Homeothermic Blanket, Harvard Bioscience, N. Natick, MA, USA).

### Videomicroscopy

Videomicroscopy of window chamber microvasculature was performed in order to directly observe tumour arterioles during experimentation (Zeiss Photomicroscope III, Carl Zeiss, New York, NY, USA). Window chamber arterioles were visualized at 200× using transillumination with either a 40 W tungsten source or mercury lamp (HBO-50W, Carl Zeiss, New York, NY, USA), depending on the thickness of the window chamber preparation. Images were acquired using a CCD camera (MTI CCD-72, Dage-MTI, Michigan City, MI, USA) and recorded on VHS videotape (SVO-9500 MD, Sony Corporation of America, San Jose, CA, USA). A videotimer image (CTG-55 Video Timer, ForA Co., Los Angeles, CA, USA) was superimposed on all video tapes for subsequent analysis of arteriolar diameter.

### Experimental protocols

Anaesthetized rats with window chambers were placed in right lateral recumbency position on the microscope stage. Following identification of the tumour feeding arterioles as described below, a laser Doppler probe was positioned beneath the stage (see below). A face mask was used to administer the various gases to the animal.

Measurements of blood pressure, heart rate, laser Doppler flow, and arteriolar diameter were taken every 30 s for the duration of the experiment. Baseline values under conditions of room air breathing were collected for a period of 20 min. The animals were then exposed to one of three different $O_2$–$CO_2$ gas mixtures for a period of 40 min. The three gas mixtures were 1% $CO_2$–99% $O_2$, 5% $CO_2$–95% $O_2$ (carbogen) and 10% $CO_2$–90% $O_2$. A baseline arterial blood gas sample under room air conditions was collected prior to the beginning of recording. A second sample was collected during exposure to the hypercarbic–hyperoxic gas at the end of experimentation.

A total of 28 rats were used in this study. Nine were exposed to 1% $CO_2$–99% $O_2$, ten were given 5% $CO_2$–95% $O_2$ and nine breathed 10% $CO_2$–90% $O_2$.

### Arteriolar diameter measurements

Tumour feeding arterioles within the subcutaneous (s.c.) vascular bed beneath the tumour were identified. The following criteria were used in defining a tumour feeding arteriole in this model system: (1) observable divergent flow; (2) straight vessel wall, with birefringence associated with the intimal layer; (3) the vessels had to be directly connected to microvessels that enter the tumour mass (Dewhirst et al, 1994). Arteriolar diameters were measured at 30 s intervals using an image shearing monitor (Model 907, IPM Inc., La Jolla, CA, USA) as described previously (Dewhirst et al, 1989).

Arteriolar diameter measurements are presented as relative data, normalized to the vessel diameter at the beginning of the experiment, i.e. time zero. The baseline diameters are presented as the average diameter during air breathing, i.e. from 0 to 20 min.

### Laser Doppler flowmetry

A single channel laser Doppler flowmeter (LaserFlow BPM 403A, TSI Inc., St. Paul, MN, USA) was used for evaluation of changes in tumour perfusion. The probe was connected to a microcomputer (Zenith Data Systems, model 2BV-3339-KQ, Benton Harbor, MI, USA) equipped with data acquisition software interfaced to digital I/O Analog output (DATAQ model DI-40). The probe was attached to the microscope stage in a fixed position, with the tip located approximately 1–2 mm beneath the window chamber

 © Cancer Research Campaign 1999

**Table 1** Arterial blood gases and arteriolar diameters (Mean (s.e.m.))

| Treatment group | *n* | pH | $P_aO2$ (mmHg) | $P_aCO2$ (mmHg) | Diameter (μm) |
|---|---|---|---|---|---|
| Room air | | 7.386 (0.002) | 90 (2) | 47.8 (0.4) | 62.7 (6.7) |
| 1% $CO_2$ | 9 | 7.368 (0.005)[a] | 494 (9)[a] | 48.6 (1.1) | |
| Room air | | 7.320 (0.019) | 103 (5) | 45.2 (2.8) | 48.5 (13.2)[c] |
| 5% $CO_2$ | 8 | 7.227 (0.036)[a,b] | 437 (23)[a,b] | 65.1 (6.1)[a,b] | |
| Room air | | 7.376 (0.006) | 85 (3) | 49.9 (0.7) | 77.7 (13.6)[d] |
| 10% $CO_2$ | 8 | 7.231 (0.006)[a,b] | 411 (5)[a,b] | 73.2 (2.7)[a,b] | |

[a]Denotes a significant difference ($P < 0.04$) between value and corresponding room air value (Wilcoxon signed-rank test). [b]Denotes a significant difference ($P < 0.05$) between value and 1% $CO_2$ value (Wilcoxon rank-sum test). [c]$n = 10$; [d]$n = 9$.

preparation. Since this device is not calibrated for measurement of absolute flow, the resultant data are reported as relative blood flow values, normalized to the flow at the beginning of the experiment, i.e. time zero. Measurements were taken every 30 s.

### Statistical methods

All experimental data were compared non-parametrically. The Wilcoxon signed-rank test was used to determine if values at any given time point were different from the baseline value (value at time zero). Differences in baseline parameters among the treatment groups were compared using the Wilcoxon rank-sum test or the Kruskall–Wallis test. All quoted *P*-values are two-sided, and significance is assumed if $P < 0.05$.

### Mathematical modelling method

The model used to analyse the effects of changes in tumour blood flow and oxygen consumption on the hypoxic fraction in tumours has been described in detail elsewhere (Secomb et al, 1995). Briefly, a Green's function method was used to calculate the distribution of oxygen tension in a region of tumour tissue containing a three-dimensional network of microvessels. The network was based on vascular architecture observed in an actual tumour growing in the dorsal flap window chamber model. The region is relatively small and does not represent the entire pathway for blood flow through a tumour. In order to include the effects of oxygen extraction from blood before it reaches the region that is simulated, we assumed that the arterial blood passed through an upstream oxygen-consuming region.

Control state values of necessary model parameters were chosen based on previous experimental observations in this preparation: arterial blood $pO_2$ = 100 mmHg, $pO_2$ of blood entering the simulation volume = 40 mmHg, and blood flow rate = $8 \times 10^{-7}$ ml $s^{-1}$. The oxygen consumption rate was set to 2.4 ml $O_2$ 100 $g^{-1}$ $min^{-1}$. By knowing these parameters, the distribution of $pO_2$ within the tissue block was determined, and the hypoxic fraction (percentage of tissue with $pO_2 < 3$ mmHg) was calculated.

The parameters used for the carbogen breathing state were also based on previous experimental observations in this preparation (Dewhirst et al, 1996): arterial blood $pO_2$ = 450 mmHg, $pO_2$ of blood entering simulation volume = 80 mmHg.

In the present study this model was used to determine the balance between $CO_2$-induced changes in blood flow and increased vascular $pO_2$ on the hypoxic fraction. For demonstration purposes, only those changes induced by carbogen breathing are presented.

### RESULTS

### Blood gases

The arterial oxygen tension ($P_aO_2$) for 25 of the 28 experiments under air breathing conditions averaged 93 ± 3 mmHg (mean ± s.e.m.). The arterial $CO_2$ tension ($P_aCO_2$) was 47.6 ± 1.0 mmHg, and the pH was 7.362 ± 0.009. Blood gas values during air breathing were not available in three rats, but the post-experiment blood gas samples verified that these animals received the hyperoxic–hypercarbic gases. There was some variability among the baseline values for the individual groups (Table 1), but most values were in the range of those seen in unanaesthetized rats (Libermann et al, 1973). The slightly high arterial $pCO_2$ is typical for rats anaesthetized with this dose of pentobarbital (Libermann et al, 1973). The arterial $pO_2$ and pH values were significantly different among the three air-breathing groups ($P < 0.03$, Kruskal–Wallis test). In particular, the air-breathing rats in the 5% $CO_2$ group were more acidotic and had higher arterial $pO_2$s than the other two groups (Table 1).

Changing the breathing gas from air to 1% $CO_2$–99% $O_2$ predictably resulted in a significant increase in $P_aO_2$ and a small significant decrease in pH (Wilcoxon signed-rank test, $P < 0.02$), but $P_aCO_2$ was not altered (Table 1). Switching the breathing gas from air to either 5% $CO_2$–95% $O_2$ or 10% $CO_2$–90% $O_2$ resulted in significant changes in all three parameters (Table 1). As might be expected, arterial pH decreased, while $P_aO_2$ and $P_aCO_2$ significantly increased ($P < 0.02$).

All three arterial blood gas parameters tended to change as the composition of the $O_2$–$CO_2$ gas mixtures was altered, although only the values during 1% $CO_2$ breathing were statistically different compared to either the 5% $CO_2$ or the 10% $CO_2$ data (Wilcoxon rank-sum test, $P < 0.05$; Table 1). As the $CO_2$ content increased and the $O_2$ content decreased, the pH and $P_aO_2$ decreased and the $P_aCO_2$ increased. The parameters measured during 5% $CO_2$ breathing were not different from those obtained during 10% $CO_2$ breathing.

© Cancer Research Campaign 1999

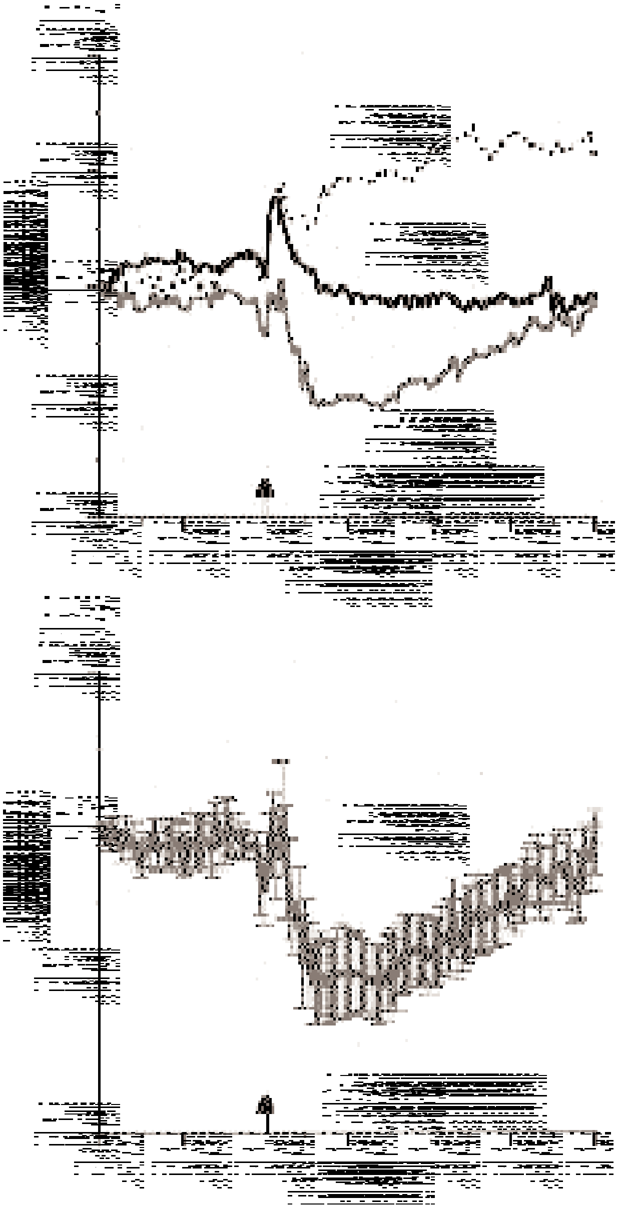

**Figure 1** Effects of breathing of hyperoxic–hypercarbic gas mixtures on relative mean arterial blood pressure (MAP) in the rat. (**A**) Rats were breathing air from 0 to 20 minutes, followed by either 1% $CO_2$–99% $O_2$ ($n$ = 9), 5% $CO_2$–95% $O_2$ ($n$ = 10), or 10% $CO_2$–90% $O_2$ ($n$ = 9) for the next 40 min. All values are relative to the MAP at 0 min and the plots are the means of $n$ rats. (**B**) Effect of 10% $CO_2$–90% $O_2$ breathing ($n$ = 9) on mean relative MAP with error bars demonstrating the standard error of the mean (s.e.m.)

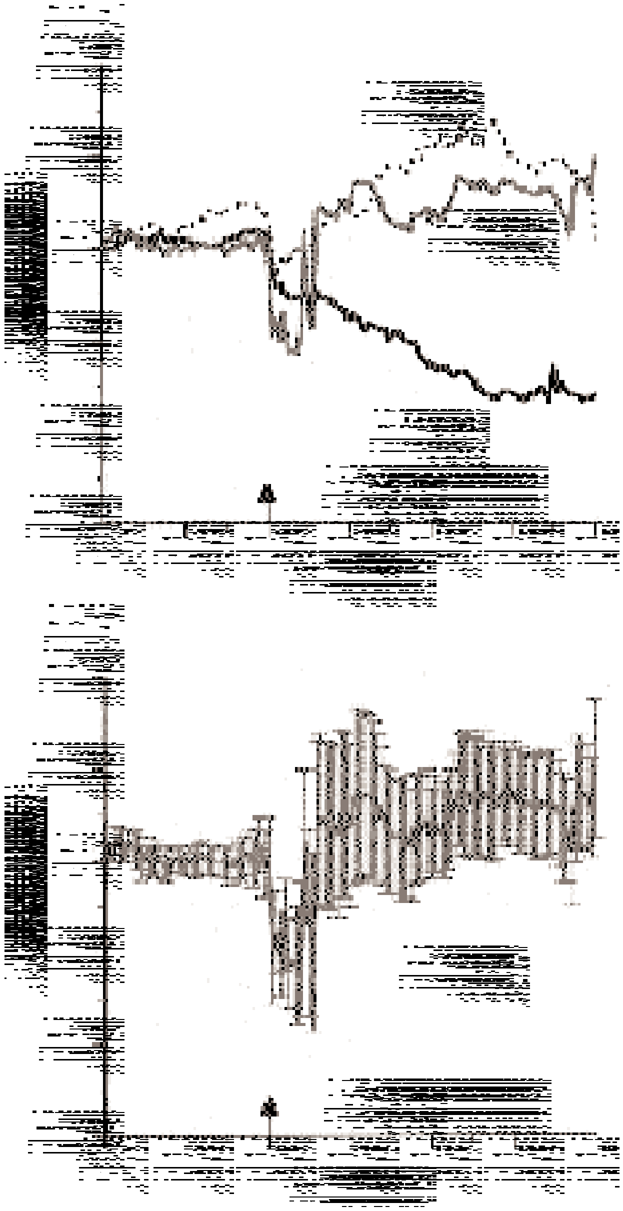

**Figure 2** Effects of breathing of hyperoxic–hypercarbic gas mixtures on relative heart rate in the rat. (**A**) Rats were breathing air from 0 to 20 min, followed by either 1% $CO_2$–99% $O_2$ ($n$ = 9), 5% $CO_2$–95% $O_2$ ($n$ = 10), or 10% $CO_2$–90% $O_2$ ($n$ = 9) for the next 40 min. All values are relative to the heart rate at 0 min and the plots are the means of $n$ rats. (**B**) Effect of 10% $CO_2$–90% $O_2$ breathing ($n$ = 9) on mean relative heart rate with error bars demonstrating the standard error of the mean (s.e.m.).

### Mean arterial pressure

Arterial blood pressures during air breathing were within normal limits for unanaesthetized rats, averaging 113 ± 2 mmHg (Smith et al, 1987). There were no significant pre-treatment differences in mean arterial pressure (MAP) among the three groups of animals ($P$ = 0.46; Kruskal–Wallis test).

Effects of gas administration on mean arterial pressure are shown in Figure 1A. Animals exposed to 1% $CO_2$ experienced an immediate 5% increase in MAP ($P$ = 0.04) that gradually increased to 10% after 20 min of gas administration and remained elevated for the duration of gas exposure ($P$ = 0.04). After an initial immediate increase in blood pressure of approximately 7% ($P$ < 0.02), animals exposed to 5% $CO_2$ experienced a sharp and sustained return to baseline values 3 min after gas administration. The effects of administration of 10% $CO_2$ on MAP are shown in Figure 1A and separately in Figure 1B, along with standard errors of the mean. Animals exposed to 10% $CO_2$ experienced a gradual decrease in MAP to almost 90% of baseline ($P$ < 0.03) 5 min after gas administration. Blood pressure remained at 90% of baseline

 *© Cancer Research Campaign 1999*

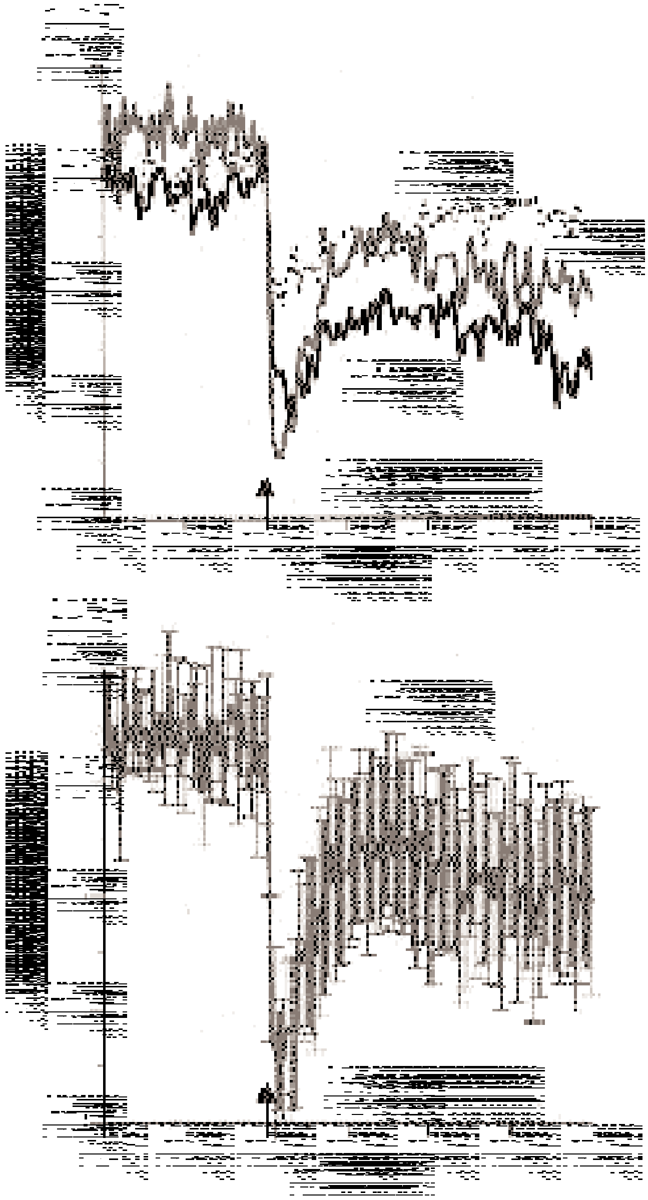

**Figure 3** Effects of breathing of hyperoxic–hypercarbic gas mixtures on relative arteriolar diameter in the rat. (**A**) Rats were breathing air from 0 to 20 min, followed by either 1% $CO_2$–99% $O_2$ ($n$ = 9), 5% $CO_2$–95% $O_2$ ($n$ = 10), or 10% $CO_2$–90% $O_2$ ($n$ = 9) for the next 40 min. All values are relative to the diameter at 0 min and the plots are the means of $n$ rats. (**B**) Effect of 10% $CO_2$–90% $O_2$ breathing ($n$ = 9) on mean relative arteriolar diameter with error bars demonstrating the standard error of the mean (s.e.m.)

values for approximately 20 min after the start of 10% $CO_2$ breathing ($P < 0.05$) before gradually returning to baseline levels after 40 min.

### Heart rate

Baseline parameters for HR were within normal limits for unanaesthetized rats, averaging 346 ± 7 beats per min (Smith et al, 1987). There were no significant pre-treatment differences in heart rate between the three groups of animals ($P$ = 0.35).

Effects of hyperoxic/hypercarbic gas administration on HR are shown in Figure 2A. Animals exposed to 1% $CO_2$ experienced no significant change in HR for the duration of gas administration. Administration of 5% $CO_2$ resulted in a trend towards an initial decrease of 3% immediately after gas exposure ($P$ = 0.09), followed by a gradual decrease of 8% at 20 min of exposure ($P < 0.04$) that was sustained throughout the duration of carbogen breathing. The effects of 10% $CO_2$ on heart rate are shown in Figure 2B, along with standard errors of the mean. Administration of 10% $CO_2$ produced no significant change in heart rate, although an initial decrease of 5% occurred immediately after gas administration and was of borderline statistical significance ($P$ = 0.06).

### Arteriolar diameter

Mean baseline arteriolar diameters during air breathing (averaged over 0–20 min) were 63, 49 and 78 μm for the 1%, 5% and 10% $CO_2$ experimental groups respectively (Table 1). Although the baseline diameters were quite variable, the values in the three different dose groups did not differ significantly from each other ($P > 0.05$).

Vasoconstriction was observed within seconds of hyperoxic/hypercarbic gas administration in all three dose groups (Figure 3A), although the statistical significance of sustained reduction in arteriolar diameter was variable. Administration of 1% $CO_2$ produced a maximal reduction in arteriolar diameter of approximately 10% 2 min after exposure ($P$ = 0.008). Carbon dioxide-induced vasoconstriction remained statistically significant for approximately 8 min after the beginning of 1% $CO_2$–99% $O_2$ breathing ($P < 0.05$). Thereafter, the diameter was not statistically different from baseline.

Administration of 5% $CO_2$ produced a maximal reduction in arteriolar diameter of approximately 20% 4 min after gas exposure ($P < 0.005$). The diameter returned to about 85% of baseline after 12 min ($P$ = 0.08), and this reduction in arteriolar diameter was sustained throughout the duration of carbogen breathing ($P < 0.05$).

The effects of 10% $CO_2$ on arteriolar diameter, along with standard errors of the mean are shown in Figure 3B. Administration of 10% $CO_2$ produced an immediate reduction in arteriolar diameter of approximately 23% ($P$ = 0.004), followed by a partial recovery toward baseline. The diameter returned to more than 90% of baseline 8 min after exposure. The vasoconstriction produced by $CO_2$ at this dose was significant only for the first 4 min of gas exposure.

### Tumour blood flow

Relative changes in TBF as assessed by laser Doppler flowmetry are shown in Figure 4A. Administration of 1% $CO_2$ produced no statistically significant change in blood flow. Breathing of 5% $CO_2$ resulted in an immediate and significant drop to approximately 65% of baseline flow after 3–4 min of gas exposure ($P$ = 0.004). Reduction in blood flow was sustained at 70–75% of baseline flow for the duration of gas exposure, with 67 of 80 time points being statistically significant ($P < 0.05$). The effects of 10% $CO_2$ on TBF along with standard errors of the mean are shown in Figure 4B. Administration of 10% $CO_2$ produced an immediate reduction to 65% of baseline blood flow after 2 min of gas exposure ($P < 0.02$). The blood flow quickly tended back toward the baseline value, and by 4 min after the beginning of 10% $CO_2$ breathing, the flow was not significantly different from baseline.

© Cancer Research Campaign 1999 

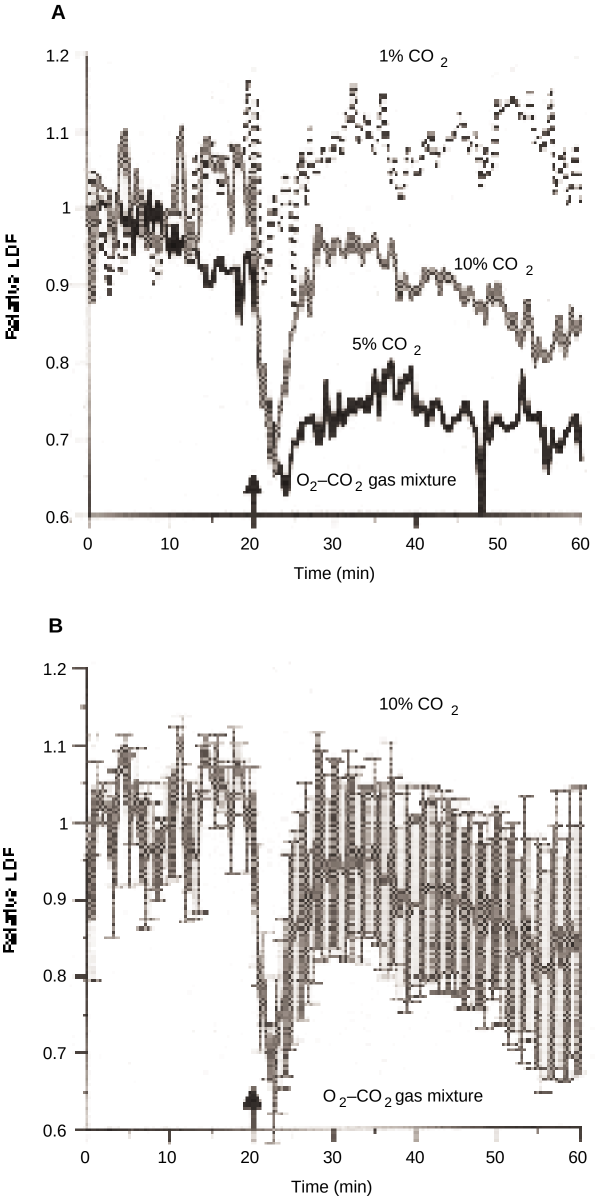

**Figure 4** Effects of breathing of hyperoxic–hypercarbic gas mixtures on relative laser Doppler blood flow (LDF) in the rat. (**A**) Rats were breathing air from 0 to 20 min, followed by either 1% $CO_2$–99% $O_2$ ($n$ = 9), 5% $CO_2$–95% $O_2$ ($n$ = 10), or 10% $CO_2$–90% $O_2$ ($n$ = 7) for the next 40 min. All values are relative to the LDF at 0 min and the plots are the means of $n$ rats. (**B**) Effect of 10% $CO_2$–90% $O_2$ breathing ($n$ = 7) on mean relative LDF with error bars demonstrating the standard error of the mean (s.e.m.)

### DISCUSSION

In this study we have altered the $CO_2$ component of an inspired hyperoxic gas in an attempt to understand and confirm previously observed changes in tumour arteriolar diameter (TAD) and TBF in the rat dorsal window chamber model. By using three different $O_2$–$CO_2$ mixtures, we tested the hypothesis that the presence of carbon dioxide in hyperoxic gases has vasoconstrictive activity in this tumour model. The effects of the three gases studied are summarized in Table 2. Breathing 1% $CO_2$–99% $O_2$ increased MAP and caused a transient tumour arteriolar vasoconstriction, but exerted no significant chronotropic or blood flow effects. Carbogen (5% $CO_2$–95% $O_2$) transiently increased MAP, decreased HR and vasoconstricted tumour arterioles, resulting in a sustained decrease in TBF. Breathing of 10% $CO_2$–90% $O_2$ caused a slight reduction in MAP but no change in HR, while transiently vasoconstricting tumour arterioles and decreasing TBF.

### Effects of different $O_2$–$CO_2$ breathing mixtures on MAP and HR

The effects of the $CO_2$ content of the breathing gases on blood pressure and HR are in general agreement with those found previously in rats (Lioy and Trzebski, 1984). Breathing 1% or 5% $CO_2$ resulted in a sustained or transient pressor response (Figure 1). On the other hand, MAP decreased transiently when the rats breathed 10% $CO_2$–90% $O_2$, which may indicate that this concentration of $CO_2$ might be sufficient to cause enough direct dilatory action of $CO_2$ on the systemic resistance vessels to dominate the response (Lioy and Trzebski, 1984). Although there was a trend for heart rate to decrease during breathing of all three hyperoxic–hypercapnic gas mixtures, the only significant change occurred during carbogen breathing, when heart rate decreased by about 8%. The $CO_2$-induced bradycardia is most likely the result of direct action of $CO_2$ on the heart (Lioy and Trzebski, 1984).

### Effects of different $O_2$–$CO_2$ breathing mixtures on TAD and TBF

As shown in Figure 3, there was a clear decrease in TAD following breathing of all hypercarbic gases. In the first minutes following the initiation of hyperoxic–hypercarbic gas breathing, the arteriolar diameter dropped significantly. There was no clear dose–response, although 1% $CO_2$–99% $O_2$ only caused a transient 10% vasoconstriction, while the initial vasoconstriction was around 20–25% after administration of the gases containing 5% $CO_2$ or 10% $CO_2$. To our knowledge, there is no other data available on the effects of breathing hyperoxic-hypercarbic gases on TAD, except for the limited data from our laboratory involving the effects of carbogen breathing (Dewhirst et al, 1996). The large decrease in arteriolar diameter seen during carbogen breathing was not evident in the earlier limited study, in which experiments were performed in only five rats, and there was a transient, but statistically insignificant, decrease in arteriolar diameter of about 10%. In re-examining those data, the mean arterial $pCO_2$ in those experiments changed only from 46 to 50 mmHg after initiation of carbogen breathing, while in this study the corresponding change was from 44 to 62 mmHg. Therefore, the more significant vasoconstriction observed in the current study may be attributable to better delivery of carbogen to the rats in this study. One question that needs to be addressed is whether the changes in TAD were the passive result of a vascular steal phenomenon or whether they were an active response to the higher $CO_2$ content of the blood. In our prior study in this model system we found that normal arterioles constricted to the same degree as tumour arterioles in response to carbogen breathing (Dewhirst et al, 1996). This argues against a vascular steal phenomenon as being responsible for the observed effects.

The changes in TBF caused by the breathing of hypercarbic gases tended to approximately mirror the changes in TAD, as shown in Figures 3 and 4. The small, transient decrease in arteriolar

© Cancer Research Campaign 1999

**Table 2** Summary of the effects of breathing of hyperoxic–hypercarbic gas mixtures on relative mean arterial blood pressure (MAP), relative heart rate (HR), relative arteriolar diameter (Diameter), and relative laser Doppler blood flow (LDF) in the rat. All values are relative to the values at 0 min and are expressed as means (s.e.m.)

| | | Time after start of 1% $CO_2$ breathing | | | |
|---|---|---|---|---|---|
| **Parameter** | ***n*** | **0** | **2** | **5** | **30** |
| MAP | 9 | 1.009 (0.028) | 1.091 (0.035)[b] | 1.053 (0.029) | 1.136 (0.040)[b] |
| HR | 9 | 1.016 (0.019) | 0.996 (0.023) | 1.005 (0.025) | 1.049 (0.041) |
| Diameter | 9 | 0.990 (0.026) | 0.887 (0.054)[a] | 0.922 (0.027)[b] | 0.984 (0.034) |
| LDF | 9 | 1.002 (0.087) | 0.939 (0.128) | 0.865 (0.117) | 1.138 (0.127) |
| | | Time after start of 5% $CO_2$ breathing | | | |
| **Parameter** | ***n*** | **0** | **2** | **5** | **30** |
| MAP | 10 | 1.011 (0.015) | 1.057 (0.017)[b] | 1.016 (0.017) | 0.989 (0.018) |
| HR | 10 | 1.009 (0.013) | 0.976 (0.013) | 0.975 (0.018) | 0.920 (0.030)[a] |
| Diameter | 10 | 1.009 (0.036) | 0.828 (0.061)[a] | 0.814 (0.055)[a] | 0.855 (0.038)[b] |
| LDF | 10 | 0.914 (0.079) | 0.724 (0.071)[a] | 0.698 (0.069)[a] | 0.726 (0.102)[b] |
| | | Time after start of 10% $CO_2$ breathing | | | |
| **Parameter** | ***n*** | **0** | **2** | **5** | **30** |
| MAP | 9 | 0.958 (0.023) | 1.005 (0.036) | 0.933 (0.018)[b] | 0.958 (0.017) |
| HR | 9 | 1.002 (0.022) | 0.965 (0.025) | 0.966 (0.050) | 1.036 (0.029) |
| Diameter | 9 | 1.029 (0.065) | 0.756 (0.052)[a] | 0.860 (0.074) | 0.942 (0.079) |
| LDF | 7 | 1.040 (0.029) | 0.698 (0.032)[b] | 0.844 (0.131) | 0.901 (0.120) |

[a]Denotes a significant difference between that value and the corresponding baseline value during air breathing at the $P < 0.01$ level based on the Wilcoxon signed-rank test. [b]Denotes a significant difference between that value and the corresponding baseline value during air breathing at the $P < 0.05$ level based on the Wilcoxon signed-rank test.

diameter following initiation of 1% $CO_2$–99% $O_2$ breathing resulted in no significant change in TBF. On the other hand, the large initial vasoconstriction caused by breathing hyperoxic gases containing 5% or 10% $CO_2$ was accompanied by a 20–30% decrease in TBF. In addition, the general trend of the recovery of the blood flow towards baseline during the breathing of these hypercarbic gases was similar to the recovery of arteriolar diameter.

Our results showing a consistent decrease in TBF during inhalation of hypercarbic gases agrees in general with the findings of Hill and co-workers (1998), who performed similar experiments in solid subcutaneously implanted sarcoma F tumours in mice. They found that any hyperoxic gas containing at least 2.5% $CO_2$ caused a decrease in TBF.

In contrast to the present results, other studies have shown either an increase or no change in TBF during breathing of hyperoxic–hypercarbic gases. Using the C3HBA mammary carcinoma implanted subcutaneously in mice as a tumour model, Kruuv et al (1966) showed that carbogen breathing resulted in a 20% increase in TBF compared to air breathing. Allowing the mice to breathe 10% $CO_2$–90% $O_2$ caused blood flow to increase by an average of 50% (Kruuv et al, 1966). Similarly, it was recently shown that carbogen breathing for 6 min resulted in a 50–70% increase in blood flow in RIF-1 tumours growing subcutaneously in mice (Honess and Bleehen, 1995). In another study, Grau and co-workers could show no carbogen-induced change in blood flow in C3H mammary carcinoma growing in the feet of mice (Grau et al, 1992). The discrepancy between these data and our current results may be due to tumour- or site-specific differences in the vasculature.

There is also a pertinent study in human patients, which examined the effects of carbogen breathing on TBF using laser Doppler technology (Powell et al, 1996). In Powell et al's study, carbogen breathing resulted in no net change in average blood flow, although there were carbogen-induced increases and decreases in TBF within the same tumour or in different tumours. In a recent study, we have examined the kinetic effects of carbogen breathing on TBF and *p*O2 in s.c. R3230Ac tumours growing in the hindlimb and in muscle sites (Lanzen et al, 1998). In the s.c. site, we observed simultaneous increases and decreases in blood flow within individual tumours, yet tumour oxygenation was unaffected in most cases. In contrast, when this same tumour line was transplanted into muscle, consistent improvements in oxygenation were observed, yet the effects on blood flow were still variable. These results all suggest that the vasoactive effects of carbogen and other hyperoxic–hypercarbic gases are complex and may be dependent upon animal model, tumour type, site of implantation or other factors.

Finally, it is interesting to compare our results directly with those of Hill and co-workers who made similar measurements of blood flow in a solid mouse tumour model during hyperoxic–hypercarbic gas breathing (Hill et al, 1998). Although they also found that TBF decreased during breathing of hyperoxic gas mixtures containing 5% or 10% $CO_2$, the decrease in blood flow followed a different time course. While blood flow in the tumour growing in the window chamber decreased almost immediately (Figure 4), blood flow in the solid mouse tumour did not significantly decrease until 30–45 min after the start of breathing the hyperoxic/hypercarbic gas. The difference in the time course of the TBF decrease may possibly be related to the mechanism behind the change. In the present study, the change in blood flow closely follows the decrease in the diameter of the tumour feeding arteriole (Figures 3 and 4), suggesting that a $CO_2$-induced vasoconstriction is directly responsible for the decrease in TBF. It has been shown that $CO_2$ can directly affect production of the vasoactive substances nitric oxide and endothelin-1, but an increase in

© Cancer Research Campaign 1999

local $CO_2$ concentration tends to lead to an increase in nitric oxide production, a decrease in endothelin-1 production and an endothelium-dependent vasodilation in most vasculature (Yoshimoto et al, 1991; Iadecola, 1992; Carr et al, 1993). Therefore, this effect of $CO_2$ could not account for the $CO_2$-induced vasoconstriction seen here. One reasonable explanation for the $CO_2$-induced vasoconstriction is suggested by the finding that some vascular beds (e.g. the mesentery) exhibit an acute vasoconstriction in response to an increase in local $CO_2$ concentration, followed by a later vasodilator effect (Nielsen et al, 1991; Carr et al, 1993). The acute vasoconstriction is independent of the endothelium (Carr et al, 1993) and is most likely mediated by an increase in the release of intracellular calcium in smooth muscle cells (Nielsen et al, 1991). In tumours, the vasoconstrictive effect of $CO_2$, resulting from direct action on vascular smooth muscle cells may dominate due to a defective or deficient endothelium-dependent vasodilatory mechanism. A similar vasoconstrictive response of tumour vasculature to the endothelium-dependent vasodilator, acetylcholine, has also been reported (Tozer et al, 1996).

In the study of Hill and co-workers, an endothelium-dependent vasodilation in normal tissue surrounding the tumours may have contributed to the observed decrease in tumour blood flow via a steal effect (Hill et al, 1998). The delay in the decrease in tumour blood flow may have been attributable to a biphasic response of the neighbouring vasculature, i.e. an initial vasoconstriction followed by a more persistent vasodilation. Therefore, the difference in TBF response to carbogen breathing in the two studies may be attributable to the sites at which they were growing. In the window chamber, the tumour was primarily supplied by the arteriole which was monitored. On the other hand, the tumour growing in the hindlimb was most likely supplied by numerous arterioles or even neovessels, whose flow could be more significantly affected by changes in the surrounding normal vascular bed.

### Theoretical estimation of effects of $CO_2$ breathing on tumour oxygenation

Secomb and co-workers (1995) have employed a mathematical model to analyse the effects of oxygen supply and demand on tumour hypoxic fraction. The Green's function model calculates hypoxic fraction based on TBF rate, blood oxygen content, tumour oxygen consumption rate and vascular geometry. The parameters used in this mathematical model were based on experimental data collected from the same experimental model used in the present study (Secomb et al, 1995; Dewhirst et al, 1996). This model was used to predict the effects of carbogen breathing on hypoxic fraction, based on the changes in TBF and arterial $pO_2$ observed during carbogen breathing. The results, which are summarized in Table 3, clearly depend on the assumptions made in setting up the model, as already described. However, this model has been found to be valuable in showing the relative sensitivity of hypoxic fraction to changes in other parameters (Secomb et al, 1995).

In the control condition utilized in this model, the hypoxic fraction (defined as the proportion of $pO_2$ measurements less than 3 mmHg) is estimated at 34%. When taking into account that carbogen reduces TBF by 30% and raises arterial $pO_2$ to 450 mmHg, the hypoxic fraction actually increases from 34% to 37%. Thus, the beneficial effect of increasing blood oxygen content is entirely negated by the decrease in TBF.

Another effect of carbogen that is often quoted as being important for enhancing tumour oxygenation is the Bohr effect, which predicts that enhanced unloading of oxygen will occur when $pCO_2$ is elevated and blood pH is reduced (Rojas, 1991). In order to consider this effect in this model, it is important to point out that prior studies in window chamber models have demonstrated that intravascular pH is relatively normal, even in regions where interstitial pH is low (Helmlinger et al, 1997). This would suggest that changes in blood gas pH would likely be reflected in the tumour microcirculation. If the Bohr effect on the oxygen–haemoglobin dissociation curve (based on a pH of 7.31 seen in the present study) is taken into account in this Green's function model, carbogen breathing reduces the hypoxic fraction from 34% to 32%. Predictions of tumour hypoxic fraction can also be made using the best case scenario, i.e. including the increase in arterial $pO_2$ seen with carbogen breathing, but assuming that TBF is not reduced. In this case the hypoxic fraction is reduced from 34% to 14%. With the same assumptions the hypoxic fraction drops from 14% to 9% if the Bohr effect is included in the calculation. The benefit realized from carbogen shifting the oxygen–haemoglobin dissociation curve does not alter tumour hypoxic fraction to a degree that would be clinically significant, since a reduction from 34% to 32% represents less than a 10% change in hypoxic fraction. This result suggests that the Bohr effect does not play a major role in affecting oxygen transport in tumours during carbogen breathing.

**Table 3** Green's function sensitivity study: effect of tumour blood flow, arterial $pO_2$, and Bohr effect on tumour hypoxic fraction

| Condition | Blood flow (%) | Arterial $PO_2$ (mmHg) | Bohr effect | Hypoxic fraction (%) |
|---|---|---|---|---|
| Control | 100 | 100 | No | 34 |
| Carbogen | 100 | 450 | No | 14 |
| Carbogen | 100 | 450 | Yes | 9 |
| Carbogen | 70 | 450 | No | 37 |
| Carbogen | 70 | 450 | Yes | 32 |

These results are consistent with our prior studies of this tumour model. Using Eppendorf oximetry we were unable to show a significant reduction in electrode hypoxic fraction during carbogen breathing (Brizel et al, 1995). In later studies we were unable to achieve any prolongation of growth time with carbogen breathing, relative to room air, after a single radiation dose of 20 Gy (Brizel et al, 1997). In a very recent study, we used microelectrodes in subcutaneous tumours to study the temporal course of oxygenation during carbogen breathing, and again found no consistent improvement in tumour $pO_2$ (Lanzen et al, 1998).

### Implications of $CO_2$ breathing on efficacy of radiation therapy

The use of carbogen as a radiosensitizer has been studied extensively in both animal and human models. In certain cases carbogen has been shown to increase tumour $pO_2$, while other studies failed to demonstrate increased TBF and oxygenation with carbogen (Song et al, 1987; Mason et al, 1991; Falk et al, 1992; Martin et al, 1993; Teicher et al, 1994; Brizel et al, 1995; Horsman et al, 1995; Powell et al, 1996, 1997; Hill et al, 1998). In some animal models, irradiation combined with carbogen breathing has demonstrated increased survival and growth delay relative to irradiation alone, while others have failed to demonstrate any benefit (Inch et al,

 © Cancer Research Campaign 1999

1970; Martin et al, 1994; Rojas et al, 1996). Despite the above evidence that carbogen might be effective in reducing tumour hypoxia, a phase III randomized human trial comparing irradiation with carbogen to irradiation without carbogen failed to demonstrate a significant survival benefit or increase in loco-regional disease control (Rubin, 1979). The reasons for the failure of carbogen to be a more effective radiosensitizer are unclear. It has been shown that carbogen's ability to increase tumour oxygen supply and radiosensitize is dependent on tissue type, pre-irradiation breathing time and route of administration (Inch et al, 1970; Siemann et al, 1977; Kallinen et al, 1991; Martin et al, 1993), suggesting that complex physiologic mechanisms are involved.

The ability of carbogen to radiosensitize more effectively than 100% $O_2$ is reportedly a result of the $CO_2$ component, which is thought to block hyperoxia-induced vasoconstriction, increase cardiac output via positive chronotropic effects and shift the oxygen–haemoglobin dissociation curve to favour the unloading of oxygen (Kruuv et al, 1996; Rojas, 1991). The results in the present study contradict the putative mechanisms by which carbogen is believed to increase tumour oxygen supply. In this experimental model we have demonstrated that carbogen breathing causes tumour arteriolar vasoconstriction, resulting in a 25–30% decrease in tumour blood flow. Mathematical models were employed to estimate the degree to which the Bohr effect would increase tumour oxygenation while breathing carbogen, and this contribution was predicted to be minimal. Thus, the decrease in tumour perfusion far outweighs any benefit gained from the increased oxygen content of the blood. These results may provide a potential explanation for the lack of consistent success in clinical trials utilizing carbogen as a radiosensitizer.

### ACKNOWLEDGEMENTS

This work was supported by a grant from the NIH/NCI CA40355 and a NATO Collaborative Research Grant 961223.

### REFERENCES

Brizel DM, Lin S, Johnson JL, Brooks J, Dewhirst MW and Piantadosi CA (1995) The mechanisms by which hyperbaric oxygen and carbogen improve tumour oxygenation. *Br J Cancer* **72**: 1120–1124

Brizel DM, Scully SP, Harrelson JM, Layfield LJ, Bean JM, Prosnitz LR and Dewhirst MW (1996) Tumor oxygenation predicts for the likelihood of distant metastases in human soft tissue sarcoma. *Cancer Res* **56**: 941–943

Brizel DM, Hage WD, Dodge RK, Munley MT, Piantadosi CA and Dewhirst MW (1997). Hyperbaric oxygen improves tumor radiation response significantly more than carbogen/nicotinamide. *Radiat Res* **147**: 715–720

Carr P, Graves JE and Poston L (1993) Carbon dioxide induced vasorelaxation in rat mesenteric small arteries precontracted with noradrenaline is endothelium dependent and mediated by nitric oxide. *Pflügers Arch* **423**: 343–345

Chapman JD, Stobbe CC, Arnfield MR, Santus R, Lee J and McPhee MS (1991) Oxygen dependency of tumor cell killing in vitro by light-activated photofrin II. *Radiat Res* **126**: 73–79

Dewhirst MW, Tso CY, Oliver R, Gustafson CS, Secomb TW and Gross JF (1989) Morphological and hemodynamic comparison of tumor and healing normal tissue microvasculature. *Int J Radiat Oncol Biol Phys* **17**: 91–99

Dewhirst MW, Madwed D, Meyer RE, Ong ET, Klitzman B, Rosner GL and Dodge R (1994) Reduction in tumor blood flow in skin flap tumor after hydralazine is not due to a vascular steal phenomenon. *Radiat Oncol Invest* **1**: 270–278

Dewhirst MW, Ong ET, Rosner GL, Rehmus SW, Shan S, Braun RD, Brizel DM and Secomb TW (1996) Arteriolar oxygenation in tumour and subcutaneous arterioles: effects of inspired air oxygen content. *Br J Cancer* **74**: S241–S246

Falk SJ, Ward R and Bleehan NM (1992) The influence of carbogen breathing on tumour tissue oxygenation in man evaluated by computerised $pO_2$ histography. *Br J Cancer* **66**: 919–924

Grau C, Horsman MR and Overgaard J (1992) Improving the radiation response in a C3H mouse mammary carcinoma by normobaric oxygen or carbogen breathing. *Int J Radiat Oncol Biol Phys* **22**: 415–419

Hampson NB, Jobsis-Vandervliet FF and Piantadosi CA (1987) Skeletal muscle oxygen availability during respiratory acid-base disturbances in cats. *Respir Physiol* **70**: 143–158

Helmlinger G, Yuan F, Dellian M and Jain RK (1997) Interstitial pH and $pO_2$ gradients in solid tumors in vivo: high-resolution measurements reveal a lack of correlation. *Nature Med* **3**: 177–182

Hickam JB and Frayser R (1966) Studies of the retinal circulation in man: observations on vessel diameter, arteriovenous oxygen difference, and mean circulation time. *Circulation* **33**: 302–316

Hill SA, Collingridge DR, Vojnovic B and Chaplin DJ (1998) Tumour radiosensitization by high oxygen content gases: influence of the carbon dioxide content of the inspired gas on $pO_2$, microcirculatory function and radiosensitivity. *Int J Radiat Oncol Biol Phys* **40**: 943–959

Höckel M, Knoop C, Schlenger K, Vorndran B, Baussmann E, Mitze M, Knapstein PG and Vaupel P (1993) Intratumoral $pO_2$ predicts survival in advanced cancer of the uterine cervix. *Radiother Oncol* **26**: 45–50

Höckel M, Schlenger K, Aral B, Mitze M, Schaffer U and Vaupel P (1996) Association between tumor hypoxia and malignant progression in advanced cancer of the uterine cervix. *Cancer Res* **56**: 4509–4515

Honess DJ and Bleehen NM (1995) Perfusion changes in the RIF-1 tumour and normal tissues after carbogen and nicotinamide, individually and combined. *Br J Cancer* **71**: 1175–1180

Horsman MR, Grau C and Overgaard J (1995) Reoxygenation in a C3H mouse mammary carcinoma. *Acta Oncol* **34**: 325–328

Iadecola C (1992) Does nitric oxide mediate the increases in cerebral blood flow elicited by hypercapnia? *Proc Natl Acad Sci USA* **89**: 3913–3916

Inch WR, McCredie JA and Sutherland RM (1970) Effect of duration of breathing 95% oxygen plus 5% carbon dioxide before x-irradiation on cure of C3H mammary tumor. *Cancer* **25**: 926–931

Kallinen J, Didier A, Miller JM, Nuttall A and Grenman R (1991) The effect of $CO_2$ and $O_2$ gas mixtures on laser Doppler measured cochlear and skin blood flow in guinea pigs. *Heart Res* **55**: 255–262

Kety SS and Schmidt CF (1948) The effects of altered arterial tensions of carbon dioxide and oxygen on cerebral blood flow and cerebral oxygen consumption of normal young men. *J Clin Invest* **27**: 484–492

Kruuv JA, Inch WR and McCredie JA (1966) Blood flow and oxygenation of tumors in mice: I. Effects of breathing gases containing carbon dioxide at atmospheric pressure. *Cancer* **20**: 51–59

Lanzen JL, Braun RD, Ong AL and Dewhirst MW (1998) Variability in blood flow and $pO_2$ in tumors in response to carbogen breathing. *Int J Radiat Oncol Biol Physics* **42**: 855–859

Libermann IM, Capano A, Gonzalez F, Brazzuna H, Garcia H and De Gelabert G (1973) Blood acid-base status in normal albino rats. *Lab Animal Sci* **23**: 862–865

Lioy F and Trzebski A (1984) Pressor effect of $CO_2$ in the rat: different thresholds of the central cardiovascular and respiratory responses to $CO_2$. *J Autonomic Nerv Syst* **10**: 43–54

Martin L, Lartigau E, Weeger P, Lambin P, Leridant AM, Lusinchi A, Wibault P, Eschwege F, Lubionski B and Guichard M (1993) Changes in oxygenation of head and neck tumors during carbogen breathing. *Radiother Oncol* **27**: 123–130

Martin LM, Thomas CD and Guichard M (1994) Nicotinamide and carbogen: relationship between $pO_2$ and radiosensitivity in three tumor lines. *Int J Radiat Biol* **65**: 379–386

Mason RP, Nunnally RL and Antich PP (1991) Tissue oxygenation: a novel determination using 19F surface coil NMR spectroscopy of sequestered perfluorocarbon emulsion and carbogen. *Magn Reson Med* **18**: 71–79

Nielsen H, Aalkjaer C and Mulvaney MJ (1991) Differential contractile effects of changes in carbon dioxide tension on rat mesenteric resistance arteries precontracted with noradrenaline. *Pflügers Arch* **419**: 51–56

Nordsmark M, Overgaard M and Overgaard J (1996) Pre-treatment oxygenation predicts radiation response in advance squamous cell carcinoma of the head and neck. *Radiother Oncol* **41**: 31–39

Overgaard J (1989) Sensitization of hypoxic tumour cells – clinical experience. *Int J Radiat Biol* **56**: 801–811

Overgaard J and Horsman MR (1996) Modification of hypoxia-induced radioresistance in tumours by the use of oxygen and sensitisers. *Semin Radiat Oncol* **6**: 10–21

Papenfuss D, Gross JF, Intaglietta M and Treese FA (1979) A transparent access chamber for the rat dorsal skin fold. *Microvasc Res* **18**: 311–38

Powell MEB, Hill SA, Saunders MI, Hoskin PJ and Chaplin DJ (1996) Effect of carbogen breathing on tumour microregional blood flow in humans. *Radiother Oncol* **41**: 225–231

© Cancer Research Campaign 1999 

Powell MEB, Hill SA, Saunders MI, Hoskin PJ and Chaplin DJ (1997) Human tumor blood flow is enhanced by nicotinamide and carbogen breathing. *Cancer Res* **57**: 5261–5264

Rojas A (1991) Radiosensitization with normobaric oxygen and carbogen. *Radiother Oncol* **20**: 65–70

Rojas A, Hirst VK, Calvert AS and Johns H (1996) Carbogen and nicotinamide as radiosensitizers in a murine mammary carcinoma using conventional and accelerated radiotherapy. *Int J Radiat Oncol Biol Phys* **34**: 357–365

Rubin P, Hanley J, Keys HM, Marcial V and Brady L (1979) Carbogen breathing during radiation therapy. *Int J Radiat Oncol Biol Phys* **5**: 1963–1970

Sampson LE and Chaplin DJ (1994) The influence of microenvironment on the cytotoxicity of TNF α in vitro. *Int J Radiat Oncol Biol Phys* **29**: 467–471

Secomb TW, Hsu R, Ong ET, Gross JF and Dewhirst MW (1995) Analysis of oxygen supply and demand on hypoxic fraction in tumors. *Acta Oncol* **34**: 313–316

Siemann DW, Hill RP and Bush RS (1977) The importance of the pre-irradiation breathing times of oxygen and carbogen (5% $CO_2$, 95% $O_2$) on the in vivo radiation response of a murine sarcoma. *Int J Radiat Oncol Biol Phys* **2**: 903–911

Smith E, Stratford IJ and Adams GE (1980) Cytotoxicity of adriamycin on aerobic and hypoxic Chinese hamster V70 cell in vitro. *Br J Cancer* **41**: 568–572

Smith TL, Coleman TG, Stanek KA and Murphy WR (1987) Hemodynamic monitoring for 24 h in unanesthetized rats. *Am J Physiol* **253**: H1335–H1341

Song CW, Lee I, Hasegawa T, Rhee JG and Levitt SH (1987) Increase in $pO_2$ and radiosensitivity of tumors by Fluosol-DA (20%) and carbogen. *Cancer Res* **47**: 442–446

Teicher BA, Schwartz GN, Dupuis NP, Kusomoto T, Liu M, Liu F and Northey D (1994) Oxygenation of human tumor xenografts in nude mice by a perfluorochemical emulsion and carbogen breathing. *Artif Cells Blood Substit Immobil Biotechnol* **22**: 1369–1375

Thomlinson RH and Gray LH (1955) The histological structure of some human lung cancers and the possible implications for radiotherapy. *Br J Cancer* **9**: 539–549

Tozer GM, Prise VE, Bell KM, Dennis MF, Stratford MRL and Chaplin DJ (1996) Reduced capacity of tumour blood vessels to produce endothelium-derived relaxing factor: significance for blood flow modification. *Br J Cancer* **74**: 1955–1960

Yoshimoto S, Ishizaki Y, Sasaki T and Murota S (1991) Effect of carbon dioxide and oxygen on endothelin production by cultured porcine cerebral endothelial cells. *Stroke* **22**: 378–383

*© Cancer Research Campaign 1999*
